# Pre-existing humoral immunity and complement pathway contribute to immunogenicity of adeno-associated virus (AAV) vector in human blood

**DOI:** 10.3389/fimmu.2022.999021

**Published:** 2022-09-16

**Authors:** Corinne J. Smith, Nikki Ross, Ali Kamal, Kevin Y. Kim, Elizabeth Kropf, Pascal Deschatelets, Cedric Francois, William J. Quinn, Inderpal Singh, Anna Majowicz, Federico Mingozzi, Klaudia Kuranda

**Affiliations:** ^1^ Immunology Department, Spark Therapeutics, Inc., Philadelphia, PA, United States; ^2^ Research Department, Apellis Pharmaceuticals, Waltham, MA, United States

**Keywords:** AAV, complement, neutralizing antibodies, innate immunity, gene therapy

## Abstract

AAV gene transfer is a promising treatment for many patients with life-threatening genetic diseases. However, host immune response to the vector poses a significant challenge for the durability and safety of AAV-mediated gene therapy. Here, we characterize the innate immune response to AAV in human whole blood. We identified neutrophils, monocyte-related dendritic cells, and monocytes as the most prevalent cell subsets able to internalize AAV particles, while conventional dendritic cells were the most activated in terms of the CD86 co-stimulatory molecule upregulation. Although low titers (≤1:10) of AAV neutralizing antibodies (NAb) in blood did not have profound effects on the innate immune response to AAV, higher NAb titers (≥1:100) significantly increased pro-inflammatory cytokine/chemokine secretion, vector uptake by antigen presenting cells (APCs) and complement activation. Interestingly, both full and empty viral particles were equally potent in inducing complement activation and cytokine secretion. By using a compstatin-based C3 and C3b inhibitor, APL-9, we demonstrated that complement pathway inhibition lowered CD86 levels on APCs, AAV uptake, and cytokine/chemokine secretion in response to AAV. Together these results suggest that the pre-existing humoral immunity to AAV may contribute to trigger adverse immune responses observed in AAV-based gene therapy, and that blockade of complement pathway may warrant further investigation as a potential strategy for decreasing immunogenicity of AAV-based therapeutics.

## Introduction

Adeno-associated viruses (AAV) are non-pathogenic viruses that display diversified tissue tropisms and are a preferred vector for delivering potentially life-changing gene therapeutics to patients in need (1). While AAV immunogenicity is relatively low, immune responses against the vector remain one of the main hurdles to successful gene therapy (2).

Individuals who have been naturally exposed to wild type AAV have ‘pre-existing’ AAV-specific neutralizing antibodies (NAbs) that cross-react with the recombinant AAV vectors used in clinic. The prevalence of anti-AAV antibodies in the human population ranges from 40-70% depending on the serotype, geographical location, or ethnicity (3–5). AAV seropositive individuals are excluded from the vast majority of AAV gene therapy trials due to predicted vector neutralization in the bloodstream and consequently lack of efficient transduction in targeted tissue (5). To overcome this issue and to allow broad access to systemic gene therapy, including AAV NAb-positive patients, strategies of pre-existing NAbs removal from circulation are being developed. These strategies include B cell depletion, plasmapheresis or using bacterial endopeptidases (6–10). Of note, while some of these approaches showed impressive efficacy to lower NAb levels and allow transduction in pre-clinical models, none of them can completely and permanently eliminate all anti-AAV antibodies. Therefore, it is important to understand whether pre-existing anti-AAV antibodies impact the innate immune response to AAV vectors, and if so to determine what levels are required to increase the risk of unwanted immune-related reactions in recipients of gene transfer.

Recently, several gene therapy trials administering high doses of systemic AAV reported cases of complement activation, occurring within a few days to 2 weeks post AAV vector infusion (11). The impacted clinical trials used different AAV serotypes, including AAV9, LK03 and C102 at doses at or above 1×10^13^ vg/kg and targeted several indications such as Duchenne muscular dystrophy (DMD), spinal muscular atrophy (SMA), methylmalonic acidemia (MMA), and Fabry disease (12–15). Reported complement activation was accompanied by thrombotic microangiopathy (TMA), which is a condition defined by the presence of thrombocytopenia, hemolytic anemia, and organ damage due to the formation of microscopic blood clots in capillaries and small arteries. When TMA is caused by complement activation it is categorized as an atypical hemolytic uremic syndrome (aHUS) (16). Currently, TMA seems to be the most frequent serious complication faced by patients receiving high-dose systemic AAV gene therapy (17). Patients with TMA following the AAV infusions often required hospitalization and treatments like, red blood cell transfusion, platelet transfusion, plasmapheresis and complement inhibitors regimens.

Complement activation can occur through three different pathways, the classical antibody-mediated pathway, the alternative pathway, and the lectin pathway. These 3 pathways converge when they produce C3 convertase enzymatic complexes that proteolytically cleave C3 and release fragments that amplify the inflammatory response and can mediate tissue injury (18). During a viral infection, complement activation leads to inflammation, opsonization, phagocytosis, and neutralization of the virus and finally results in activation of the adaptive immune response (19). In particular, complement may reduce Treg function (20, 21) and promote T and B cell activation (22, 23). Complement components have been also shown to bind directly AAV capsid, increasing cytokine/chemokine gene expression and vector uptake in monocytic cell lines or primary murine bone marrow macrophages. *In vitro*, AAV activated complement through the classical antibody-mediated pathway, while complement receptors were involved in anti-AAV antibody formation in mice (24).

In this study, we showed that AAV NAb titers ≥1:100 significantly increased innate immune response to AAV vectors, namely, the uptake of AAV by various blood phagocytes and dendritic cells, pro-inflammatory cytokine release, complement activation and C3 split products deposition on neutrophils. Furthermore, we tested APL-9, a pegylated derivative of compstatin (25) that has an identical mode of action as APL-2 (EMPAVELI™), recently approved for the treatment of paroxysmal nocturnal hemoglobinuria. Using APL-9, we demonstrated that AAV uptake, AAV-induced dendritic cells activation and cytokine release were dependent on having a functional complement pathway. These results show that complement is an important modulator of the anti-AAV immune response in human blood, and that inhibition of the complement pathway may potentially be a promising strategy for preventing anti-AAV immune responses in clinic.

## Materials and methods

### AAV vectors

The AAV vectors were produced in roller bottles by triple transfection of HEK293 cells, purified and concentrated by cesium chloride gradient purification as previously described (10). QC testing included purity check by SDS-PAGE/Silver stain, genomic titer by qPCR, and sterility tests including bioburden, endotoxin and mycoplasma. AAV-Spark100 (highly homologous to AAV8) and AAV-LK03 (highly homologous to AAV3B) vectors expressed Gaussia luciferase under the control of the CAG (CMV early enhancer/chicken β actin) promoter. For the comparison of cytokine secretion in response to full or empty vector, we used AAV-Spark100 expressing human FIX40 and empty AAV-Spark100 particles purified from the same vector preparation. The percentage of full and empty AAV particles in vector preparations was determined by Stunner (Unchained Labs, CA) analyzer that combines ultraviolet–visible (UV–Vis) spectroscopy, static light scattering (SLS), and dynamic light scattering (DLS) (26). The material designated as ‘full’ contained 84.7 ± 3.3% of AAV particles with DNA cargo. The separated ‘empty’ fraction contained 90.6 ± 0.3% of AAV particles void of DNA.

### Human blood collection

Whole blood was collected from healthy human donors in sodium citrate coated tubes and used for whole blood stimulation assays on the same day (within 8 hours of collection). Donor demographics and anti-AAV neutralizing antibody titers are detailed in **Supplemental Table 1**. Sodium citrate was chosen because it largely preserves the function of the complement cascade up through C3 cleavage (27). The choice of anticoagulant is an important one when studying complement and although sodium citrate is known to possess some complement inhibitory properties, particularly toward the classical and lectin pathways, its effect on the complement system is less than other anticoagulants such as heparin, EDTA or EGTA (27). All human specimens were procured under IRB approved protocols from consented donors by StemExpress, BioIVT or Flowmetric.

### Whole blood assay

200μL of undiluted whole blood was distributed into 96 well plates and AAV was added to a final concentration of 1×10^11^ vg/mL or 5×10^11^ vg/mL. Where indicated, APL-9 was added at a final concentration of 2.4μM. As a positive control, blood was incubated with a protein pool of cytomegalovirus, influenza and parainfluenza protein antigens (CPI) at 1mg/mL (CTL, Cat # CTL-CPI-001). Negative control wells were treated with vector vehicle (PBS with 0.001% Pluronic) in a volume equal to the volume of AAV added to the treatment wells. Blood was incubated at 37°C in a 5% humidity incubator. All conditions were tested in duplicate or triplicate. Supernatants were carefully collected from whole blood after centrifugation at 90 minutes (for C3a ELISA) and 24 hours (for MSD) time points post treatment.

### NAb assay

Neutralizing antibody titers against AAV-Spark100 and LK03 were determined using a cell-based luciferase reporter assay as described previously (28) with modifications. Serum samples were serially diluted in FBS and incubated with AAV-Spark100 or AAV-LK03 vectors expressing gaussia luciferase (GLuc) for 30 min at 37°C. The AAV-serum mix was then applied to freshly seeded 2V6.11 cells in a 96-well tissue culture plate. GLuc in the supernatant was collected and measured 24 hours after transduction using the Renilla Luciferase Assay System (Promega, Cat #E2829) kit with luminescence detection by a GloMax Discover plate reader (Promega). The antibody titer was determined as the highest serum dilution which inhibited transduction by 50% (IC50).

### Cytokine and chemokines

Levels of pro-inflammatory cytokines IFN-γ, IL-1β, IL-2, IL-4, IL-6, IL-8, IL-10, IL-12p70, IL-13, and TNF-α were measured using the human V-PLEX Proinflammatory Panel 1 kit from Meso Scale Diagnostics (MSD, Cat #K15049D-1) and chemokine levels for Eotaxin, Eotaxin-3, IL-8 (HA), IP-10, MCP-1, MCP-4, MDC, MIP-1α, MIP-1β, and TARC were measured using the human V-PLEX Chemokine Panel 1 kit from Meso Scale Diagnostics (MSD, Cat #15047D-1). Assays were run according to the manufacturer’s instructions. Briefly, plates were washed three times with provided wash buffer prior to adding samples. Samples were diluted 1:1 in sample dilution buffer provided and added to the 10-spot V-PLEX plates in duplicate along with serially diluted calibrator controls of known concentration for each analyte. Samples and controls were incubated at room temperature with shaking at 550 rpm. Following sample incubation, plates were washed three times with wash buffer and then incubated with detection antibodies conjugated to an electro-chemiluminescent SULFO-TAG for 1 hour at room temperature with shaking. Plates were then washed three times with wash buffer. Read Buffer T (2X) was added, and plates were read immediately by the Meso Sector S 600 (Meso Scale Diagnostics) plate reader. Cytokine and chemokine concentration was interpolated from the calibration control standard curve by the Meso Scale Discovery Workbench 4.0 software.

### Flow cytometry

For detection of C3 fragments by flow cytometry, the cells remaining from the previously described whole blood assay were collected and surface stained with a monoclonal antibody to human-C3d (clone BGRL 11, ARP) and other phenotypic markers. For staining of cells for AAV uptake, 1mL of fresh whole blood was plated in 12-well plates. AAV-LK03 was added to a final concentration of 5×10^11^ vg/mL and when indicated APL-9 was added at a final concentration of 2.4μM. At 24 hours post treatment, red blood cells were lysed with ACK buffer. Adherent cells were dislodged by incubating in PBS with 5μM EDTA and scraping with a cell scraper. Cells were stained for surface markers HLA-DR (clone Tu36, Biolegend), CD11b (clone ICRF44, Biolegend), CD86 (clone IT2.2, Biolegend), CD123 (clone 6H6, Biolegend), CD14 (clone M5E2, Biolegend), CD19 (clone HiB19, Biolegend), CD16 (3GB, Biolegend) and CD11c (clone 3.9, Biolegend), and then fixed and permeabilized with the eBioscience FoxP3 Transcription Factor Staining buffer set according to the manufacturer’s instructions. Cells were stained for intracellular AAV overnight at 4° using the biotin-conjugated A20R clone (Progen). Cells were subsequently stained intracellularly with a fluorochrome labelled streptavidin secondary for 1 hour at room temperature. All samples were run on a LSR Fortessa (BD Biosciences) and analyzed using FlowJo V10.6 (FlowJo LLC).

### Complement C3 ELISA

C3a sandwich ELISA kits were purchased from Quidel and used in accordance with the manufacturer’s protocols (Quidel, Cat #A031). Briefly, supernatant or serum samples were thawed in 37°C water bath and immediately diluted 1:400 in the provided sample dilution buffer. Samples were added to the C3a microassay plate in duplicate and incubated at room temperature for 1 hour followed by three washes with the provided wash buffer. Following washing, C3a conjugate was added to each well, incubated at room temperature for 1 hour, and washed again three times. Next, substrate detection reagent was added to each well for 15 minutes at room temperature followed by stop solution to halt the enzymatic reaction. The C3a microassay plate was read immediately at 450 nm using a Spectramax i3 plate reader (Molecular Devices). Data was analyzed using Prism software (Graphpad).

### Complement inhibition

The pegylated compstatin analogue APL-9 was kindly provided by Apellis Pharmaceutical, Inc. For *in vitro* experiments, APL-9 was reconstituted in 5% dextrose and used at a final concentration of 2.4μM. APL-9 was added to samples immediately prior to addition of vector.

## Results

### Identification of cellular subsets in human blood able to internalize AAV vector

**Figure 1:**
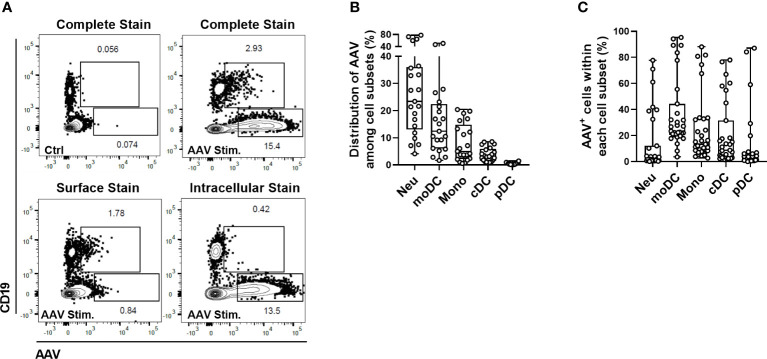
Identification of cellular subsets in human blood able to internalize AAV vector. Blood cells were stimulated with 5×10^11^ vg/mL of AAV-LK03 for 24 hours. **(A)** Representative FACS plots that show intracellular localization of AAV particles in CD19^-^ cells and surface localization on the CD19^+^ cells. **(B)** Distribution of AAV^+^ cells among blood cell types. **(C)** Percent of AAV^+^ cells within each cellular subset.

When AAV vectors are administered intravenously, the first contact between viral particles and the host immune system occurs in the peripheral blood. Phagocytic cells in the blood can internalize viral particles to avoid the systemic spread of infectious agents and are an important part of the innate defense against viruses. Here we sought to identify the cellular subsets that interact with AAV vectors in circulation before they can reach and transduce targeted tissue. To this end, we optimized an assay able to detect intracellular AAV particles before the intracellular degradation of the capsid protein occurs. AAV-LK03 was added for 1.5, 6 or 24 hours to blood and AAV particles were detected by intracellular staining using the A20 antibody (Progen). Intracellular staining of AAV was the most prominent at 24 hours (**Supplemental Figure 1**) and this time-point was used for further sample analysis. To verify that the staining truly represented internalization of AAV and not just sticking of vector to the cell surface, control staining was performed *i.* without cell permeabilization (exclusive surface staining) or *ii*. with an unlabeled anti-AAV antibody to block surface AAV detection added prior to cell permeabilization and intracellular staining with labeled anti-AAV antibody (exclusive intracellular staining). Using this method, we found that B cells (CD19^+^) were positive for AAV staining exclusively on the cell surface, while the remaining CD19^-^ cells contained only intracellular vector (**Figure 1A**). When cells were further phenotyped (gating strategy and naming conventions adapted from (29, 30) and shown in **Supplemental Figure 2**), we found that among total live cells most AAV-positive cells were neutrophils (29.76% ± 21.76) and monocyte-derived dendritic cells (moDCs) (16.27% ± 13.30), followed by monocytes (8.15% ± 6.95), conventional dendritic cells (cDCs) (3.79% ± 2.61) and plasmacytoid dendritic cell subsets (pDCs) (0.48% ± 0.39) (**Figure 1B**). While a large proportion of the AAV^+^ cells in the blood were neutrophils, less than 15% of total neutrophil population contained detectable vectors (**Figure 1C**). This result may reflect the higher frequency of circulating neutrophils, rather than their preferential uptake of the vector. In contrast, ~35% of all moDCs and ~25% of all monocytes were positive for intracellular AAV staining (**Figure 1C**).

### Activation of conventional dendritic cells is observed upon AAV uptake

**Figure 2:**
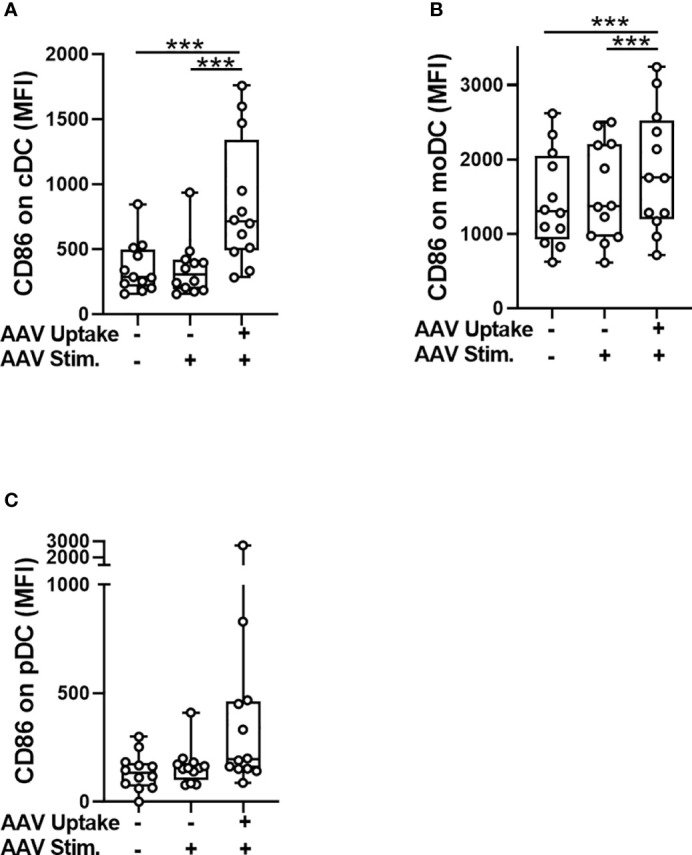
Activation of conventional dendritic cells (cDC) coincides with the AAV uptake. Whole blood from healthy human donors was stimulated with AAV-LK03 at 5×10^11^ vg/mL for 24 hours and stained for intracellular AAV and phenotypic markers. Mean fluorescence intensity (MFI) of CD86 staining is shown for **(A)** cDC, **(B)** moDC and **(C)** pDC. Symbols represent values of each donor. Significance was determined with One-way ANOVA with Tukey’s multiple comparisons test. *** p ≤ 0.001.

The internalization of AAV by blood phagocytes may increase secretion of pro-inflammatory cytokines (24, 31) and could also potentially reduce the amount of vector reaching a desired target tissue following systemic AAV delivery. Additionally, some phagocytes, such as dendritic cells, can present viral antigens to the immune cells to further initiate adaptive immune response. To test whether the uptake of AAV causes the activation of dendritic cells in blood, we measured cell surface levels of CD86 expression which is not only an activation marker, but also plays a critical role in T-cell priming (32). Flow cytometry analysis found a modest but statistically significant increase of CD86 levels in cells that contained intracellular AAV (AAV^+^) compared to AAV-negative (AAV^-^) or unstimulated control cells (**Figures 2A-C**). The CD86 upregulation in response to AAV was mostly pronounced in the cDC cell subset (2.6-fold compared to controls, **Figure 2A**). These results suggest that cDC might become activated upon AAV uptake and in theory mediate the priming of AAV-specific adaptive immune response, as has been already suggested in mice (33).

### AAV NAbs enhance uptake of AAV vector into cDC and other blood phagocytes

Consistent with previous studies, (3–5) the blood donors in this study had a range of naturally occurring, pre-existing antibodies to AAV (**Supplemental Table 1**). To understand the role of pre-existing anti-AAV antibodies in the innate immune response to AAV vectors, we stratified blood samples into seronegative (NAb <1:1), low seropositive (NAb ≤1:10) or high seropositive (NAb ≥1:100) groups and measured vector uptake in different cellular subsets using flow cytometry.

**Figure 3:**
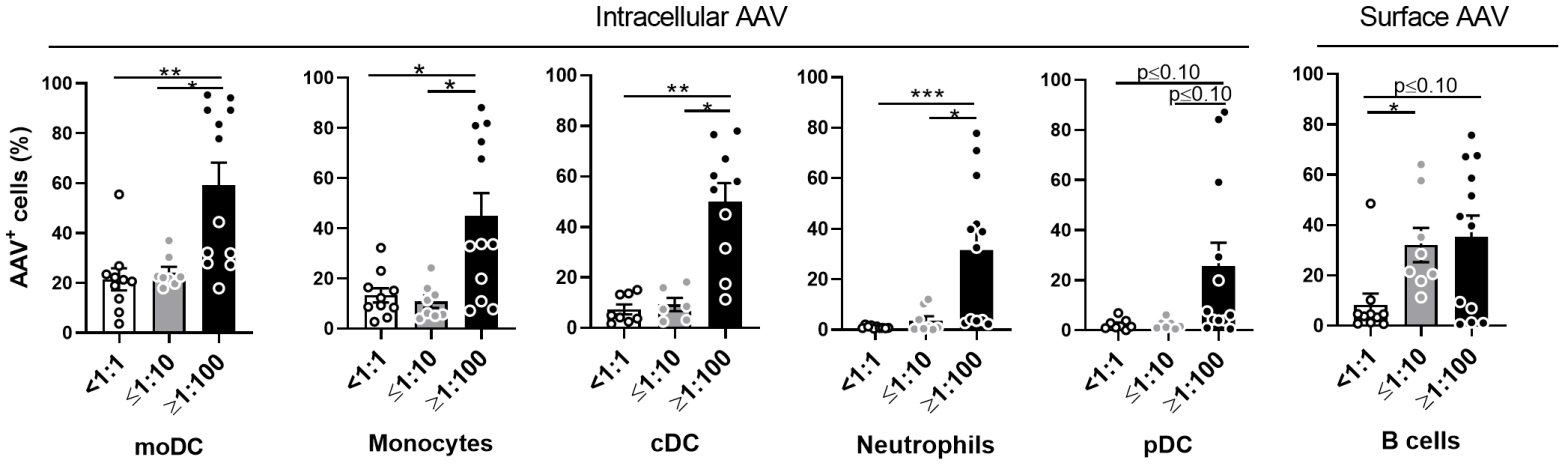
AAV NAbs enhance uptake of AAV vector into blood phagocytes. Whole blood from healthy human donors was stimulated with AAV-LK03 at 5×10^11^ vg/mL for 24 hours and stained for intracellular AAV and phenotypic markers. Samples were categorized based on their titers of anti-AAV-LK03 neutralizing antibodies as indicated under x axis. Graphs show percentage of AAV^+^ cells within the indicated cell subtype. **Figure 1C** shows data from the same set of samples. Bars represent the mean ± SEM and symbols represent the individual values of blood samples. Significance was determined by the Kruskal-Wallis test with Dunn’s multiple comparisons test. *p ≤ 0.05; ** p ≤ 0.01; *** p ≤ 0.001;.

Except for pDCs, the presence of high NAb titers significantly increased vector uptake in all tested cell types (**Figure 3**). To further verify which cell type could internalize the highest quantity of vector per single cell, we compared the mean fluorescence intensity (MFI) of AAV staining in AAV^+^ cells of different cellular subsets. The intensity of staining in all cellular subsets of seronegative blood samples were similar. In the high NAb titer donors, there was statistically higher MFI of AAV staining in moDC, cDC, neutrophils, monocytes, and B cells (**Supplemental Figure 3**). As expected, these results suggest that in NAb-positive blood the uptake of vector by immune cells will be more efficient, compared to seronegative blood.

### AAV NAbs enhance anti-AAV pro-inflammatory cytokine response in blood

**Figure 4:**
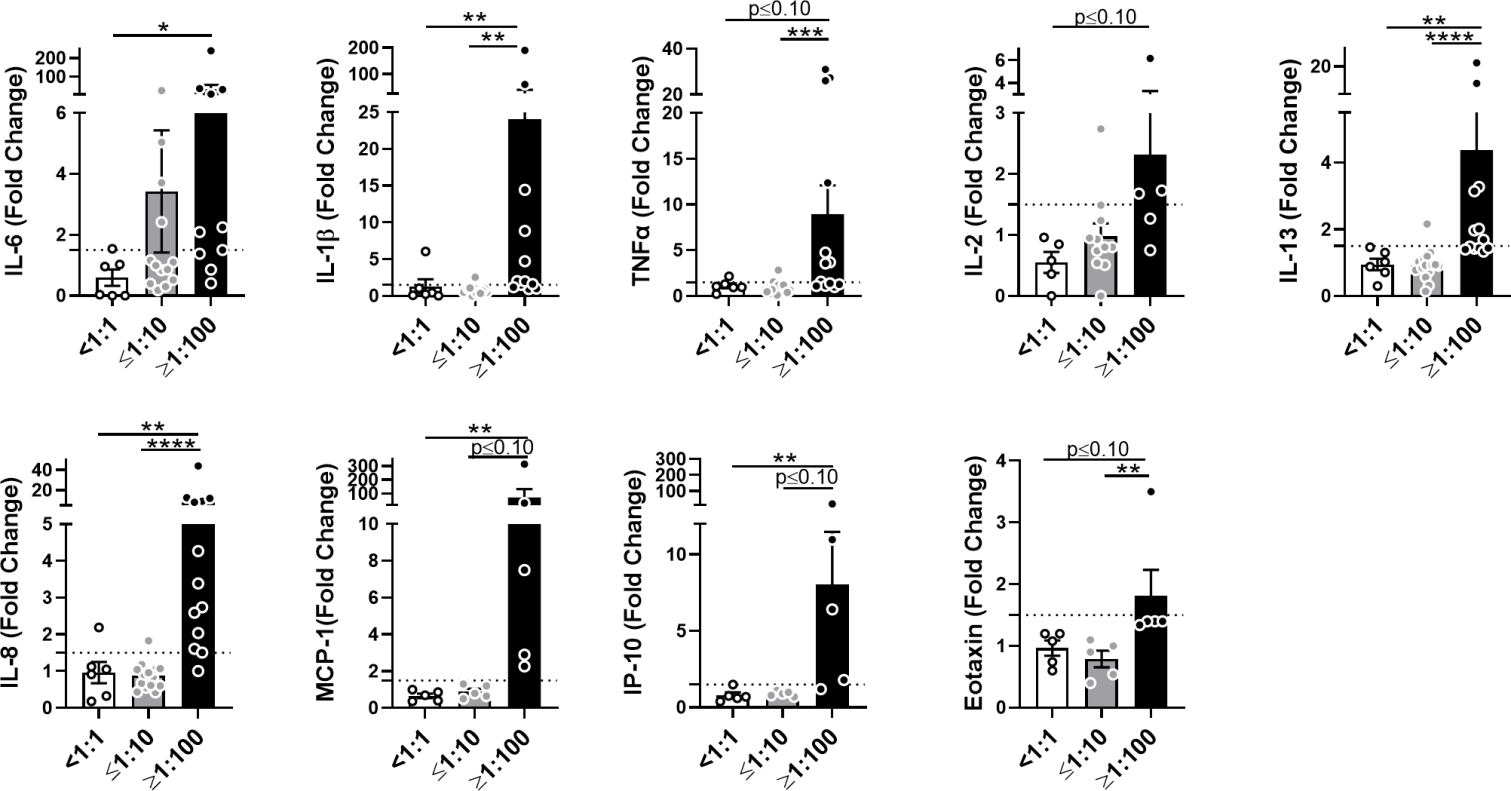
AAV NAbs enhance anti-AAV pro-inflammatory cytokine response in blood. Cytokine and chemokine secretion from whole blood stimulated with 5×10^11^ vg/mL of AAV-Spark100 for 24 hours. Samples were categorized based on their titers of anti-AAV-Spark100 neutralizing antibodies as indicated under x axis. Bars represent mean fold-change ± SEM of each analyte’s concentration measured in AAV-stimulated relative to unstimulated blood. Symbols represent the values of each donor. Dashed lines signify a 1.5-fold positivity cutoff. Significance was determined by the Kruskal-Wallis test with Dunn’s multiple comparisons test. *p ≤ 0.05; **p ≤ 0.01; ***p ≤ 0.001; ****p ≤ 0.0001.

To assess the early cytokine response to AAV in human blood, we incubated samples from healthy donors containing various titers of AAV-Spark100-neutralizing antibodies (NAbs) with the AAV-Spark100 vector. Twenty-four hours later we measured secreted cytokines and chemokines using the MSD platform. Levels of IFN-γ, IL-4, IL-10, IL-12p70, Eotaxin-3, MCP-4, MDC, MIP-1β or TARC were not significantly affected by AAV exposure or NAb presence (**Supplemental Figure 4**). In contrast, the secretion of IL-6, IL-1β, TNF-α, IL-2, IL-13, IL-8, MCP-1, IP-10, and Eotaxin in response to AAV was significantly increased in high NAb blood, but not in seronegative blood (**Figure 4** and **Supplemental Figure 5**). We found that NAb-negative samples (<1:1) rarely showed a response more than 1.5-fold higher when compared to unstimulated cells (**Figure 4**). These results demonstrate a magnified anti-AAV immune response in the blood of individuals previously exposed to naturally occurring wild-type AAV compared to seronegative individuals.

### AAV NAbs enhance AAV-induced complement activation and C3 split products deposition on neutrophils

The central component of the complement pathway, C3, is cleaved to generate C3a anaphylatoxin and C3b opsonin (18). C3a is then quickly converted into C3a-desArg that can be measured by ELISA. At the same time, C3b breaks down to iC3b, C3dg and C3d that opsonize viral particles to neutralize them or bind complement receptors on immune cells, for instance neutrophils (18). The binding of C3b and its split products on the cell surface can be detected using flow cytometry (34).

**Figure 5:**
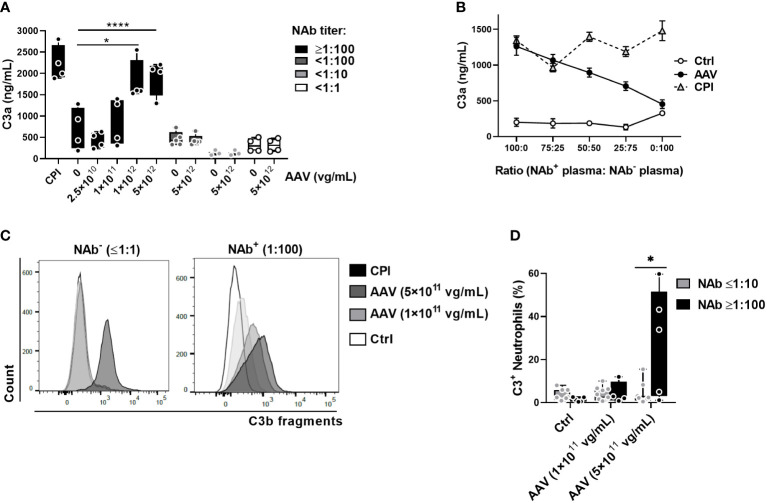
AAV NAbs enhance AAV-induced complement activation and C3 split products deposition on neutrophils. **(A)** C3a levels in human plasma with the indicated NAb titers, treated for 1 hour with various concentrations of AAV-Spark100. Significance was determined with a two-way ANOVA with Sidak’s multiple comparisons test, *p ≤ 0.05, ****p ≤ 0.0001. **(B)** NAb^-^ (<1:1) and NAb^+^ (≥1:100) plasma was mixed at the indicated ratios and stimulated with 5×10^11^ vg/mL of AAV-Spark100. Mean C3a levels ± SEM are shown (n=8). **(C)** Representative FACS plots and **(D)** graph of C3 fragment deposition on CD11b^+^ cells in the blood at 90 minutes after treatment with the indicated concentrations of AAV-Spark100. Bars represent the mean ± SEM and symbols represent the values of individual donors. Significance determined using the Holm-Sidak method *p ≤ 0.05.

Here, we first used C3a-desArg ELISA to test serum and plasma samples with various NAb titers and spiked with several AAV-Spark100 vector concentrations. We found that in plasma (**Figure 5A**) as well as in serum and whole blood (**Supplemental Figures 6A, B**), AAV-Spark100 activated complement only when NAbs were present at levels ≥1:100. Similar results were obtained with AAV-LK03 (**Supplemental Figure 6C**). Observed level of C3a increase is potentially biologically relevant, since less than 1.5-fold increase of C3a is observed in acute phase of thrombotic thrombocytopenic purpura (form of TMA) in human (35). Three-fold C3a increase is observed in patients with dengue hemorrhagic fever compared to infected but not ill patients (36).

Finally, it is known that circulating levels of complement proteins can differ considerably between individuals (37), thus our results could be affected by this variability rather than the NAb titers. To rule out variability in the ability of AAV- Spark100 NAb-high samples to activate complement independently of NAbs, we mixed NAb-positive plasma with different ratios of NAb-negative plasma. We found that, while CPI activated complement to a similar degree in all mixtures, an increased proportion of the AAV-Spark100 NAb-positive plasma always showed higher complement activation (**Figure 5B**).

Interestingly, AAV-induced activation of complement observed in highly NAb-positive whole blood samples coincided with increased deposition of C3b fragments on neutrophils as measured by flow cytometry (**Figures 5C, D**). It has been previously shown that cell bound C3 fragments on neutrophils increased response to cytokine stimulation, enhanced neutrophil degranulation, and oxidative burst (34).

Together these data demonstrate that, *in vitro*, AAV-Spark100 and -LK03 activate complement through the antibody-dependent process. These results suggest that AAV administration in the presence of anti-capsid antibodies may result in higher levels of complement activation.

### Empty capsid particles trigger complement activation and pro-inflammatory cytokine secretion

**Figure 6:**
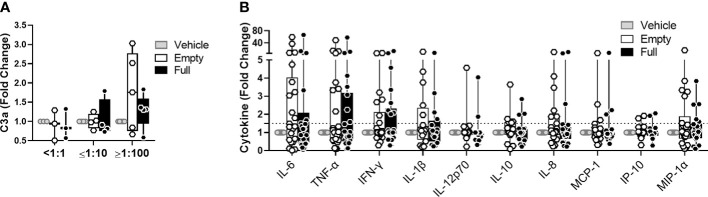
Empty vectors induce similar response to full vectors in whole blood assay. **(A)** C3a levels in human serum treated with 5×10^11^ vg/mL of AAV-Spark100-GLuc ‘full’ or 5×10^11^ of AAV-Spark100 ‘empty’ capsid/mL or vector vehicle for 90 minutes. **(B)** Cytokine and chemokine secretion from whole blood stimulated with 1×10^11^vg/mL of AAV-Spark100-hFIX ‘full’ or 1×10^11^ AAV-Spark100 empty capsid/mL or vector vehicle for 24 hours. Samples were categorized based on their titers of anti-AAV-Spark100 neutralizing antibodies as indicated under x axis. Bars represent mean fold-change ± SEM of AAV relative to unstimulated cells. Symbols represent values of each sample. Dashed lines signify a 1.5-fold positivity cutoff. Significance was determined with the Holm-Sidak method and showed no significant changes.

Empty capsid particles, void of DNA cargo, are a common contaminant of AAV vector preparations used in clinical trials and in theory increase the overall AAV antigen load received by recipients of gene transfer. We sought to understand to what extent empty capsids can contribute to early immune stimulation. To this end, we compared a similar quantity of full and empty AAV-Spark100 particles, obtained from the same vector preparation, and their potential to activate complement in human serum or to activate cytokine release in the blood. In this setting, complement activation (**Figure 6A**) and cytokine secretion (**Figure 6B**) in response to empty AAV particles was comparable to that seen with full vectors. This suggests that the capsid protein alone is a sufficient trigger of early immune responses to AAV.

### C3 complement inhibition lowers innate immune response to AAV in blood

**Figure 7:**
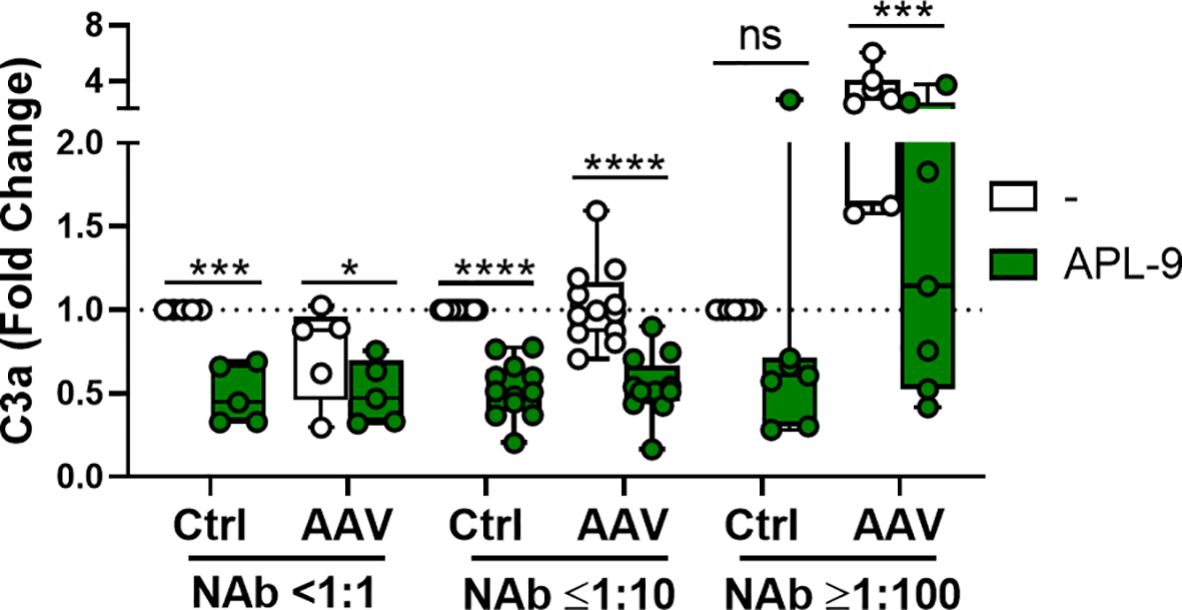
APL-9 lowers complement activation in NAb-positive and negative blood. C3a levels in whole blood measured 90 minutes post AAV-Spark100 (5×10^11^ vg/mL) addition with or without APL-9. Bars represent the mean fold change ± SEM relative to unstimulated cells. Symbols represent the individual values in samples. Significance was determined with a two-way RM-ANOVA with Sidak’s multiple comparison test. *p ≤ 0.05; ***p ≤ 0.001; ****p ≤ 0.0001. ns, not significant.

Since the complement pathway is a very potent mediator of inflammation, we tested whether inhibition of the pathway could lower immune response to AAV in human blood. To this end, we used a C3 complement inhibitor, APL-9, a derivative of compstatin that blocks complement C3 hydrolysis and can bind several C3 split products (25). We stimulated human whole blood with 5×10^11^ vg/mL of AAV-Spark100 for 90 minutes in the presence or absence of APL-9. We found that APL-9 not only lowered the AAV-induced complement activation in NAb-high samples, but also significantly lowered the basal levels of C3a in NAb-low and NAb-negative samples (**Figure 7**).

**Figure 8:**
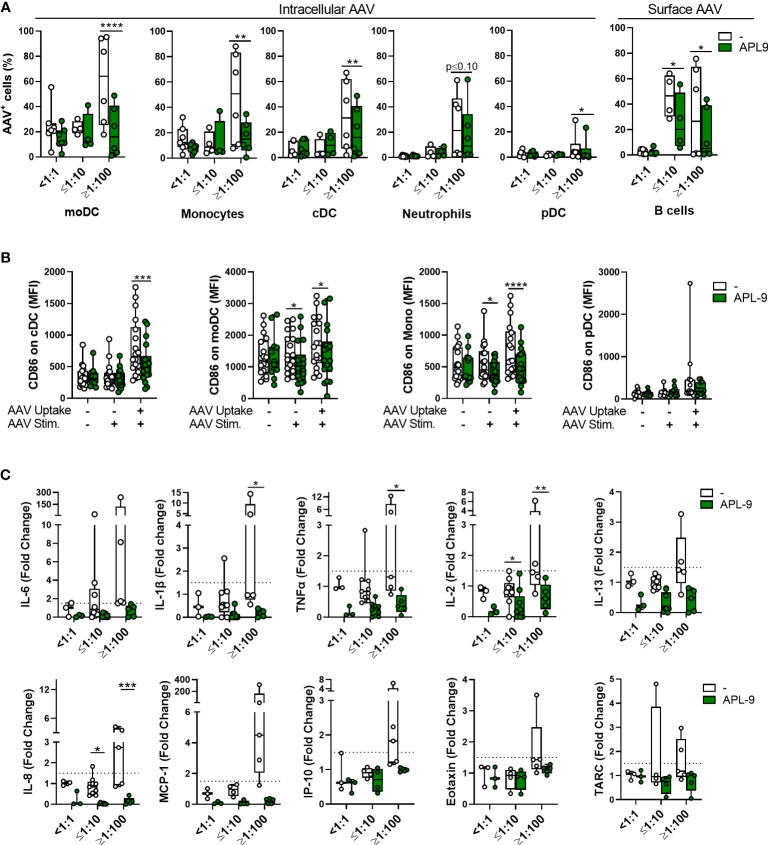
C3 complement inhibition lowers innate immune response to AAV in blood. Whole blood from healthy human donors was stimulated with 5×10^11^ vg/mL of AAV-LK03 with or without APL-9 for 24 hours and stained for intracellular AAV and phenotypic markers. **(A)** Percentage of AAV^+^ cells within the indicated cell subtype. Bars represent the mean ± SEM and symbols represent the individual values of blood samples. Significance was determined with a two-way RM-ANOVA with Sidak’s multiple test comparison. **(B)** Mean fluorescence intensity (MFI) of CD86 staining for the indicted cell type. Significance was determined with a mixed effects analysis with Sidak’s multiple comparisons test. **(C)** Cytokine and chemokine secretion from whole blood stimulated with 5×10^11^ vg/mL of AAV-Spark100 for 24 hours. Samples were categorized based on their titers of anti-AAV-Spark100 neutralizing antibodies as indicated under x axis. Values for control group are duplicated from **Figures 2**–**4**. Bars represent mean fold-change ± SEM of each analyte’s concentration measured in AAV-stimulated relative to unstimulated blood. Symbols represent the values of each donor. Dashed lines signify a 1.5-fold positivity cutoff. Significance was determined with a 2way ANOVA with Sidak’s multiple comparisons test. *p ≤ 0.05; **p ≤ 0.01; ***p ≤ 0.001; ****p ≤ 0.0001.

Next, using flow cytometry, we found that APL-9 significantly reduced vector uptake for all tested cellular subsets and AAV binding to B cells, in NAb-high samples. There was also a trend towards lower AAV uptake in APL-9-treated moDCs and monocytes from NAb-negative donors, although it did not reach statistical significance (**Figure 8A**). Further, in monocytes, cDCs, and MoDCs that had taken up vector, despite the presence of APL-9, the surface levels of CD86 were lower compared to controls (**Figure 8B**). Finally, the secretion of multiple pro-inflammatory cytokines or chemokines was also lowered by APL-9 treatment, which was the most significant in NAb high blood samples (**Figure 8C**).

Together, these data suggest that complement plays a significant role in the AAV uptake by phagocytes, in particular by APCs, such as cDC and moDC, as well as their activation. Complement pathway activation also promotes pro-inflammatory cytokine and chemokine response to AAV.

## Discussion

The initial clinical studies of AAV-based gene transfer targeting liver correlated the AAV-specific cytotoxic T lymphocytes activation with transaminitis and loss of transgene expression, therefore studies of AAV immunogenicity focused mainly on the adaptive immune responses against AAV-transduced hepatocytes (38, 39). Given these clinical manifestations of AAV immunogenicity, the goal of commonly used immunomodulation regimes was to inhibit T cell function and included mostly corticosteroids, sirolimus, tacrolimus, mycophenolate mofetil, or cyclosporine (40, 41). More recent work has highlighted the role of innate immune system in response to AAV therapeutics (42–46) further supported with the reports of complement-mediated TMA cases in subjects receiving high doses of systemic AAV (40, 41)

Here, using human blood *in vitro*, we demonstrate that complement and pre-existing AAV neutralizing antibodies play a crucial role in the innate response to AAV vectors. We have identified neutrophils, moDC and monocytes, as the most numerous innate immune cell subsets in human blood able to take up AAV. We have also shown that at the single cell level vector uptake coincided with the increased CD86 levels on the cell surface. This effect was the most pronounced in cDCs, followed by moDCs. Both of these dendritic cell types have been previously implicated in responses to AAV. In mice, cDCs were shown to play a role in priming of a cytotoxic T cell response against immunogenic ovalbumin peptide inserted into the AAV2 capsid protein sequence (42). moDCs have been previously identified as the main cells secreting IL-6 and IL-1β in response to AAV in human blood (43), or activation of T follicular helper T cells in the mouse model of muscle gene transfer (47, 48). A different type of dendritic cells, pDCs, was shown to secrete type I interferons in response to AAV (49), but did not participate directly in CD8^+^ T cell priming (42). This response was mediated by TLR9 and dependent on MyD88. In our study, we have not measured type I interferon levels in blood stimulated with AAV, but on the average only ~2% of pDC shown intracellular staining for AAV and the CD86 levels in cells containing AAV were not significantly changed. This was in contrast with ~37% of all moDCs and ~21% of all cDCs that were at the same time positive for intracellular AAV staining.

Our work shows that the presence of pre-existing AAV NAbs strongly enhance the innate immune responses to the vector, namely, the uptake of AAV by various blood phagocytes and dendritic cells, pro-inflammatory cytokine and chemokine release, complement activation and C3 split products deposition on neutrophils. We observed a high donor-to-donor variability in the degree of cytokine/chemokine secretion and vector uptake after stimulation of blood with AAV, even in donors with comparable NAb titers, likely due to heterogeneity in the antibody subclasses and function (50, 51) or different complement levels that vary in each individual or with age, (37). Nevertheless, this is an important finding particularly given that in some cases AAV vectors may be administered in the presence of anti-capsid antibodies which could result in more frequent immune-related toxicities compared to NAb-negative patients and require more targeted immunosuppressive treatments. To this aim, using the C3 inhibitor APL-9, we demonstrated significant lowering of cytokine and chemokine secretion in blood, as well as the uptake of vector and activation of APC, in blood from both seronegative and seropositive donors. Moreover, a general decrease of uptake of AAV by all blood phagocytes was observed, which could lead to higher availability of vector to the targeted tissue in systemic gene transfer applications and therefore dose sparing. These findings are consistent with what is known about the complement function. Importantly, the complement system plays a key role in viral peptides presentation by APCs to activate antigen-specific T cells. This happens through the opsonization, which improves antigen recognition and uptake into APCs, upregulation of costimulatory molecules on APCs and induction of pro-inflammatory cytokine secretion (31, 52, 53). Thus, inhibiting complement could potentially help lowering or avoiding adaptive immune responses to the vector. Complement inhibition is an attractive strategy to lower AAV immunogenicity as it is more targeted than corticosteroids, which would also address complement-related toxicities observed in some trials. Complement activation is one of the earliest events in an innate immune response, and therefore early intervention at that point could help mitigate multiple downstream effector functions for both the innate and adaptive immune response. While this study focused on the innate immune response to AAV, complement split products have been shown to be critical for T cell activation, proliferation and survival (54) (52), reducing Treg function (18), lowering the activation threshold for B cells (55) and have been shown to delay the humoral immune response and significantly lower titers of neutralizing antibodies in response to AAV in mice (24). Therefore, complement inhibition may become an attractive candidate as an immunosuppressive strategy for gene therapy. Last, preventing phagocytosis of vector by immune cells may affect the biodistribution *in vivo* by allowing more vectors to reach the target tissue, which could permit successful gene therapy with lower doses of vector.

In this study we also found benefits of inhibiting complement in donors that did not have a pronounced complement response to AAV and were NAb-negative. Under physiologic conditions, low levels of C3 are spontaneously hydrolyzed, even in the absence of a specific complement-activating stimulus (56). APL-9 reduced this basal level of complement activation in blood and decreased AAV-induced cytokine/chemokine secretion in NAb-negative samples. Indeed, it has been previously shown that C3 complement components can bind to the AAV capsid even in the absence of antibodies, and that this can subsequently act as an opsonin that facilitates phagocytosis and the production of cytokines from APCs (24). In addition, there is an increasing body of evidence that local activation of complement can have autocrine and paracrine functions that are independent of the systemic activation of the complement cascade. Many cells of the immune system – including polymorphonuclear cells, mast cells, monocytes, macrophages, dendritic cells, T cells and B cells - are capable of secreting complement components and some also secrete enzymes that can activate the pathway locally (57). Therefore, the benefits of inhibiting complement in gene therapy patients may be twofold, *i*, preventing acute toxicity caused by systemic complement activation and *ii*, reducing the contribution of background levels of complement activation to AAV immunogenicity. This supports the potential utility of inhibiting complement in gene therapy regardless of the total dose of vector infused or the serological state of the patient.

Our study and earlier work by Zaiss et al. (24) showed that complement activation by AAV *in vitro* strictly depends on the presence of anti-AAV antibodies. In theory, this observation would fit the timing of complement activation observed in clinical trials, around one week post vector infusion, which could coincide with *de novo* developed anti-AAV antibodies and relatively high quantity of vector still present in circulation. However, certain preclinical (58, 59) and clinical (13) studies suggested the alternative pathway activation following systemic AAV infusion in seronegative animal models or humans. Our study does not address this question, although measuring complement activation in blood *in vitro* may miss the important components of this response *in situ* such as hepatocytes and Kupffer cells. For instance neutrophils, identified here as the main cells able to internalize AAV in blood and binding C3 on the cell surface in response to AAV, not only store properdin (60), a critical positive regulator of the alternative pathway (61), but were also shown to infiltrate mouse liver one hour after intravenous vector infusion. This transient effect was abolished by the Kupffer cell depletion (62). Further investigation is needed to clarify whether complement activation *in vivo* is strictly dependent on anti-AAV antibody presence.

A relevant implication of this work is the observed interaction of the innate immune system with AAV empty capsids. It is known that immunogenicity of AAV and the incidence of immune-related adverse events increases with the vector dose [reviewed (63)]. AAV doses are calculated based on determination of vector genomes so-called ‘full’ capsids, however, depending on the manufacturing method or batch-to-batch variation, empty capsids can correspond to 50%–90% of the total generated AAV particles (64). In theory, empty AAV particles might significantly increase the total viral antigen load without providing a therapeutic benefit to the patient. In this study, we have found that empty vector particles were able to elicit complement activation and cytokine secretion similarly to full AAV particles. This is consistent with our results that complement activation in this setting was mainly dependent on the interaction of the capsid protein with AAV-specific antibodies. Genome presence did not significantly increase these responses, but it should be noted that for cytokine measurement we used a single stranded DNA and largely CpG depleted. It is likely that the difference would be more pronounced if more immunogenic DNA would be used, for instance CpG-rich or self-complementary (65–69). Importantly, these results suggest that high levels of empty capsids in AAV preparations may contribute to complement activation.

In conclusion, AAV vectors interact with the human innate immune system and particularly the complement. Our results suggest that the pre-existing humoral immunity to AAV vectors may be an important determinant in the immune safety of AAV-based gene therapy and that blockade of complement pathway may be a promising strategy for decreasing immunogenicity of AAV-based therapeutics.

## Data availability statement

The raw data supporting the conclusions of this article will be made available by the authors, without undue reservation.

## Ethics statement

The studies involving human participants were reviewed and approved by IRBs at StemExpress, BioIVT and Flowmetric. The patients/participants provided their written informed consent to participate in this study.

## Author contribution

CS, WQ and KlK designed research studies. CS, NR, AK, KeK, EK conducted experiments. CS, IS, AM, FM, KlK prepared and revised the manuscript. PD and CF provided APL-9 and reviewed the manuscript. All authors contributed to the article and approved the submitted version.

## Acknowledgments

We thank JoAnne Coleman for assistance with statistical analysis and Dave Derosa for technical support with ELISAs and MSD. Results shown in **Figures 1**-**5** were performed as a part of ARDAT project. The ARDAT project has received funding from the Innovative Medicines Initiative 2 Joint Undertaking under grant agreement No 945473. This Joint Undertaking receives support from the European Union’s Horizon 2020 research and innovation program and EFPIA. This communication reflects the views of the authors and neither the IMI nor the European Union, EFPIA or any other partners are liable for any use that may be made of the information contained herein.

## Conflict of interest 

CS, NR, AK, KeK, EK, IS, AM, FM, and KlK are employees of Spark Therapeutics. PD and CF are employees of Apellis Pharmaceuticals. WJQ was an employee at Spark Therapeutics at the time that the study was conducted, but is no longer.

## Publisher’s note

All claims expressed in this article are solely those of the authors and do not necessarily represent those of their affiliated organizations, or those of the publisher, the editors and the reviewers. Any product that may be evaluated in this article, or claim that may be made by its manufacturer, is not guaranteed or endorsed by the publisher.
