# Supplementary material for: Pre-existing humoral immunity and complement pathway contribute to immunogenicity of adeno-associated virus (AAV) vector in human blood
Source: Front Immunol. 2022 Sep 16;13:999021. doi: 10.3389/fimmu.2022.999021 (PMC9523746; doi:10.3389/fimmu.2022.999021)
Supplement: Supplementary file 1 [file DataSheet_1.docx]

Supplemental Tables

**Table 1:** Donor demographics and anti-AAV neutralizing titers for donors used in all figures excluding Figure 6.

| **Donor ID#** | **Sex** | **Age** | **Race** | **NAb (Spark100)** | **NAb (LK03 )** |
| --- | --- | --- | --- | --- | --- |
| EN01000209 | Female | 45 | Caucasian | <1:1 | <1:1 |
| EN01000171 | Female | 38 | Caucasian | 1:1 | <1:1 |
| EN01000269 | Female | 20 | Caucasian | 1:1 | <1:1 |
| EN01000253 | Female | 19 | Caucasian | 1:10 | <1:1 |
| EN01000234 | Female | 25 | Caucasian | <1:1 | <1:1 |
| EN01000242 | Female | 53 | Caucasian | <1:1 | <1:1 |
| EN01000038 | Male | 46 | Caucasian | <1:1 | <1:1 |
| EN01000304 | Male | 25 | Caucasian | 1:1 | <1:1 |
| EN01000229 | Male | 20 | Caucasian | <1:1 | <1:1 |
| HMN750983 | Male | 35 | Hispanic | 1:1 | <1:1 |
| 20938 | Male | 44 | Hispanic | <1:1 | <1:1 |
| EN01000232 | Male | 41 | Caucasian | 1:1 | <1:1 |
| EN01000266 | Male | 28 | Caucasian | <1:1 | <1:1 |
| EN01000261 | Female | 21 | African American | 1:1 | 1:1 |
| EN01000217 | Female | 38 | African American | 1:1 | 1:1 |
| EN01000231 | Female | 31 | Caucasian | 1:1 | 1:1 |
| RR02122 | Male | 59 | African American | 1:10 | 1:1 |
| EN01000273 | Female | 43 | Caucasian | 1:1 | 1:10 |
| EN01000252 | Male | 47 | Unspecified | 1:10 | 1:10 |
| RR02702 | Male | 32 | Hispanic | 1:1 | 1:10 |
| 44870 | Male | 44 | Hispanic | 1:1 | 1:10 |
| EN01000238 | Male | 41 | Caucasian | 1:1 | 1:10 |
| EN01000270 | Female | 20 | Caucasian | >1:100 | >1:100 |
| EN01000295 | Female | 44 | Caucasian | >1:100 | >1:100 |
| EN01000281 | Female | 59 | Caucasian | 1:10 | >1:100 |
| EN01000228 | Female | 23 | Caucasian | >1:100 | >1:100 |
| RR02094 | Female | 68 | Caucasian | 1:1 | >1:100 |
| EN01000194 | Male | 67 | Caucasian | >1:100 | >1:100 |
| 56763 | Male | 38 | Caucasian | >1:100 | >1:100 |
| 31624 | Male | 65 | Hispanic | >1:100 | >1:100 |
| RR00145 | Male | 29 | Hispanic | >1:100 | >1:100 |
| 52812 | Male | 32 | Hispanic | >1:100 | >1:100 |
| 50107 | Male | 51 | African American | >1:100 | >1:100 |
| EN01000204 | Male | 57 | African American | >1:100 | >1:100 |

**Supplemental Figures**

**Supplemental Figure 1. AAV uptake time course.** Representative FACS plots of AAV-LK03 uptake at different time points in human whole blood.

**Supplemental Figure 2: Gating strategy and cell phenotype for whole blood assay.** A) Representative FACS plots and B) Phenotypic markers that were used to define B cells, conventional dendritic cells (cDC), classical monocytes (referred to as monocytes or Mono), plasmacytoid dendritic cells (pDC), neutrophils (Neu) and monocyte-related dendritic cells (MoDC) in the whole blood assay.

**Supplemental Figure 3: Mean fluorescence intensity of vector in vector positive cells.** Vector uptake in human whole blood stimulated with full AAV-LK03 at 5×10^11^ vg/mL for 24 hours. Graphs show MFI of vector in AAV^+^ cells within the indicated cell subtype. Bars represent the mean, error bars represent the SEM and symbols represent the values of individual donors. Significance was determined by One way ANOVA with Tukey's multiple comparisons test. *p≤0.05, ** p≤0.01 *** p≤0.001, **** p≤0.0001.

**Supplemental Figure 4:** Cytokine and chemokine secretion from whole blood stimulated with AAV-Spark100 at 5×10^11^ vg/mL for 24 hours. Donors were categorized based on their titers of AAV-Spark100 neutralizing antibodies. Bars represent mean fold-change of AAV relative to unstimulated cells. Error bars represent SEM. Symbols represent the values of each donor. Dashed lines signify a 1.5-fold positivity cutoff. Significance was determined by the Kruskal-Wallis test with Dunn's multiple comparisons test. *p≤0.05, ** p≤0.01, *** p≤0.001, **** p≤0.0001

**Supplemental Figure 5:** Cytokine and chemokine secretion from whole blood stimulated with AAV-Spark100 at 5×10^11^ vg/mL or vehicle for 24 hours. Donors were categorized based on their titers of AAV-Spark100 neutralizing antibodies. Bars represent mean pg/mL of the indicated cytokine. Error bars represent SEM. Symbols represent the values of each donor. Significance was determined by mixed effects analysis with Tukey's multiple comparisons test. *p≤0.05, ** p≤0.01, *** p≤0.001, **** p≤0.0001

**Supplemental Figure 6: Complement activation in human serum and whole blood.** A) C3a levels in human serum treated with 5×10^11^ vg/mL of AAV-Spark100 in the presence or absence of APL-9 for 1 hour. Significance was determined with a 2way ANOVA with Sidak's multiple comparisons test. B) C3a levels in whole blood treated with the indicated dose of AAV- Spark100 for 90 minutes. Significance was determined with a mixed effects analysis with Dunnet’s multiple comparisons test. C) human serum treated with AAV-LK03 full or empty vectors at the indicated concentrations in donor with different NAb titers. Significance was determined with a 2way ANOVA with Sidak's multiple comparisons test. *p≤0.05; ** p≤0.01; *** p≤0.001; **** p≤0.0001.
